# Lens-based wavefront sensorless adaptive optics swept source OCT

**DOI:** 10.1038/srep27620

**Published:** 2016-06-09

**Authors:** Yifan Jian, Sujin Lee, Myeong Jin Ju, Morgan Heisler, Weiguang Ding, Robert J. Zawadzki, Stefano Bonora, Marinko V. Sarunic

**Affiliations:** 1 School of Engineering Science, Simon Fraser University, Burnaby, BC, Canada; 2 UC Davis RISE Small Animal Ocular Imaging Facility, Department of Cell Biology and Human Anatomy, University of California Davis, Davis, CA 95616, USA; 3 Vision Science and Advanced Retinal Imaging laboratory (VSRI), Department of Ophthalmology & Vision Science, University of California Davis, Sacramento, CA 95817 USA; 4 CNR-Institute for Photonics and Nanotechnology, Via Trasea 7, 35131, Padova, Italy

## Abstract

Optical coherence tomography (OCT) has revolutionized modern ophthalmology, providing depth resolved images of the retinal layers in a system that is suited to a clinical environment. Although the axial resolution of OCT system, which is a function of the light source bandwidth, is sufficient to resolve retinal features at a micrometer scale, the lateral resolution is dependent on the delivery optics and is limited by ocular aberrations. Through the combination of wavefront sensorless adaptive optics and the use of dual deformable transmissive optical elements, we present a compact lens-based OCT system at an imaging wavelength of 1060 nm for high resolution retinal imaging. We utilized a commercially available variable focal length lens to correct for a wide range of defocus commonly found in patient’s eyes, and a novel multi-actuator adaptive lens for aberration correction to achieve near diffraction limited imaging performance at the retina. With a parallel processing computational platform, high resolution cross-sectional and *en face* retinal image acquisition and display was performed in real time. In order to demonstrate the system functionality and clinical utility, we present images of the photoreceptor cone mosaic and other retinal layers acquired *in vivo* from research subjects.

Adaptive optics (AO) was first introduced for astronomy to sharpen the images acquired of the stars from terrestrial telescopes^1^. With the AO technique, the optical aberrations due to inhomogeneity in the atmosphere were measured with a wavefront sensor, and then corrected using a deformable mirror controlled in a closed feedback loop.

Recently, the AO concepts have been merged with microscopy techniques, resulting in the advancement of high resolution biological imaging. More details about this exciting topic can be found in a recent review^2^. In particular, AO has been successfully applied to cellular resolution imaging of the retina, enabling visualization of the characteristic mosaic patterns of the outer retinal layers using flood illumination fundus photography^3^,^4^, scanning laser ophthalmoscopy (SLO)^5^,^6^,^7^, and optical coherence tomography (OCT)^8^,^9^,^10^,^11^,^12^,^13^. In the case of human retinal imaging, the cornea and intraocular lens combined act as the objective lens, so that the maximum attainable lateral resolution on the retina is related to the diameter of the dilated pupil. Imaging with a narrow diameter beam using infrared light, about 1–2 mm in diameter as with common commercially available retinal diagnostic system, results in a spot size on the retina of nominally 25 μm in diameter. As the diameter of the imaging beam at the cornea increases, thus increasing the numerical aperture (NA), the effect of optical aberrations in the refractive elements of the eye also increases, which results in blurring the focal spot. Using AO to correct the wavefront distortions enables diffraction limited imaging up to the maximum NA of the human eye about 0.23 with a 8 mm beam diameter at the pupil. Despite the high quality of the *in vivo* images attainable with the AO and its undisputable clinical and basic science value, there has been a limited uptake of this technology into a clinical ophthalmology.

The exquisite resolution afforded by the AO comes at the price of a limited field of view and specialized equipment. Recent reports in the literature describe efforts to address the limitations of AO-SLO in the clinic^14^. The typical integration of the AO with an ophthalmic imaging system results in a relatively large and complicated optical setup. The wavefront measurement is commonly performed using a Hartmann-Shack Wavefront Sensor (HS-WFS) placed at an image plane that is optically conjugated to the pupil of the eye. The deformable mirror is placed at an optical plane conjugated to both the pupil and the HS-WFS. Due to the high sensitivity of the HS-WFS to the back-reflections, the optical system is commonly constructed with concave mirrors instead of lenses. In order to reduce the aberrations from the off-axis use of spherical mirrors, long focal lengths are desired, consequently increasing the size of the system. Out-of-plane configurations of optical elements have also been proposed to further minimize aberrations^15^,^16^.

We present a novel lens-based AO-OCT retinal imaging system with significant potential to overcome many of the barriers to integration with a clinical environment. In order to reduce the system size and complexity, we propose the use of a wavefront sensorless adaptive optics (WSAO) algorithm to drive the shape of the wavefront correcting element based on an image quality metric. By eliminating the HS-WFS, the WSAO approach is compatible with typical lens-based optical systems, which can be readily designed with a simpler and smaller footprint than the mirror-based AO systems while maintaining diffraction limited imaging performance. In addition, instead of the deformable mirror, we propose to use a novel transmissive multi-actuator adaptive lens (MAL) capable of correcting aberrations up to 4^th^ radial order Zernike polynomial; the MAL design and operation has been fully described in detail in our recent publication^17^. A benefit of using the transmissive wavefront correcting element is that it can be placed adjacent to a pre-defined pupil plane without the need for an extra optical relay, thus further reducing the footprint of the optical system.

Practical implementation of the MAL-WSAO-SS-OCT in a clinical environment also requires high speed OCT image acquisition and processing capabilities. We integrated the WSAO algorithm with our comprehensive OCT acquisition and massively parallel processing platform^18^,^19^. Real-time volumetric display and feature tracking of the OCT cross-sectional (B-scan) retinal images permitted depth resolved aberration correction with the WSAO algorithm. In practice, an arbitrary *en face* retinal depth-plane of interest (i.e. a particular retinal cell layer) could be selected by the operator and used as the input to a merit function for WSAO aberration correction. A swept source (SS) OCT engine in the 1010–1110 nm wavelength range, close to the transmission window of water (and the vitreous humour in the eye), was selected to reduce the effects of scattering in the ocular anterior chamber, and provide deeper tissue penetration in the retina. The SS-OCT also provided a combination of rapid depth scan (A-scan) rate and high sensitivity over a long image depth (low signal roll-off), permitting the full thickness of the retina to be visualized while keeping the high lateral resolution even with a short depth of focus^20^.

In the remainder of this report, we describe in detail the implementation of a compact lens-based MAL-WSAO-SS-OCT, and present *in vivo* retinal imaging results acquired from human volunteers. Wide-field of View OCT images of the retina were used for visualizing the cross-sectional structures of the retinal layers in B-scan images, and for navigating across the retina based on anatomical landmarks (e.g. blood vessels) in *en face* images. Narrow-field of View images were used for visualizing the photoreceptor mosaic *en face* at various retinal eccentricities from the fovea. The potential benefits and limitations of the MAL-WSAO-SS-OCT system for clinical retinal imaging are discussed.

## Methods

### Adaptive optics retinal SS-OCT imaging system

**Figure 1:**
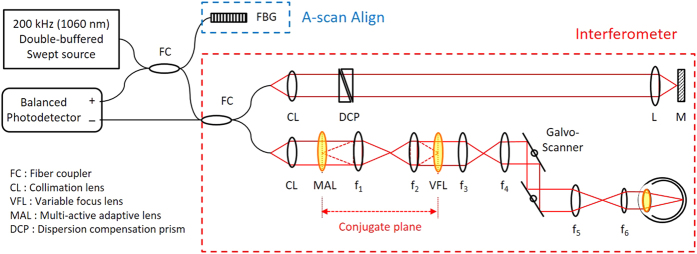
Schematic diagram of MAL-WSAO-SS-OCT system. CL, 37.5 mm; f_1_ = 200 mm, f_2_ = 50 mm, f_3_ = 50 mm, f_4_ = 50 mm, f_5_ = 100 mm, f_6_ = 200 mm.

Figure 1 shows a schematic of the MAL-WSAO-SS-OCT system. A wavelength swept laser (Axsun Inc.) with a center wavelength of 1060 nm and a full width at half maximum (FWHM) of 80 nm, double-buffered to a 200 kHz A-scan rate, was used as a light source for the OCT system; a detailed description of double-buffered swept source engine is provided in ref. 20. The light from the source was split by an optical fiber coupler and then directed to the interferometer built with single-mode fibers and a Fiber Bragg Grating used to generate an A-scan alignment signal^20^. In the interferometer, one port of the fiber coupler was connected to a sample arm for delivering light to the eye, as described in Section 2.2, and the other port was directed to a reference arm. The interference signal was detected by a 350 MHz balanced photodetector (PDB430C, Thorlabs Inc.). The signals from the detector were then sampled by a digitizer (ATS9350, AlazarTech Inc.) with 12-bit resolution and a sampling rate of 500 MHz.

### Adaptive optics light delivery to the eye

The sample arm of the MAL-WSAO-SS-OCT system consisted of the MAL (research prototype designed and built by Dr. Stefano Bonora), variable focus lens (VFL, ARCTIC 316-AR850, Varioptic), relay lenses, and galvanometer mounted mirrors to deliver a scanning beam to pupil of the subject being imaged. The MAL was placed next to the first lens collimating the light from the fiber. This optical plane was optically conjugated to the VFL via an optical relay. Two additional relay optics were used to conjugate the optical plane to the two galvanometer mounted mirrors, and then to the pupil of subject. Based on the Gullstrand-LeGrand model of the human eye^21^, the average focal length of the eye and theoretical NA were determined to be 22.67 mm and 0.2 respectively with 4.8 mm beam diameter at the pupil and refractive index of 1.33 for a water at 1060 nm, which results in a 1/e^2^ waist of 2.40 μm at the retina (corresponding to FWHM spot size of 2.83 μm). The dual deformable elements (MAL and VFL) created a basic ‘woofer-tweeter’ like adaptive optics system^22^,^23^,^24^. The key function of the VFL was to accommodate for the variation in the eyes of subjects up to approximately −4.8 diopters without any mechanical adjustment of the lenses positions. The axial position of the focus within the retina was readily observed as the location of bright focal band seen at retinal layers changed dynamically as the control voltage applied to the VFL was varied. All of the other lenses used in the system were standard achromatic doublets (Thorlabs Inc. and Edmund Optics Inc.). The total length of the sample arm was 1.5 m, and was folded to fit on an optical breadboard (0.61 m × 0.46 m) which was mounted to a slit lamp base (Haag Streit), providing three dimensional translation of the imaging system relative to the eye of subject. During human retinal imaging, the widest field of view used was ~6° × 6°, which is equivalent to retinal area of 1.8 mm × 1.8 mm.

### Aberration correction

The MAL was used for higher order aberration correction as well as fine tuning of the focus. Although the MAL is capable of correcting aberrations up to 4^th^ order Zernike polynomials, we restricted the aberration correction modes to those with highest impacts for the 5 mm beam at the pupil, as described in ref. 25. The modes that we used were defocus, 0 deg and 90 deg astigmatisms and 0 deg and 90 deg comas, which are corresponding to Zernike modes of 4, 3, 5, 6, and 7 in the OSA standards for reporting the aberrations of the eye^26^. The generalized WSAO optimization algorithm we employed has been previously described^27^,^28^, but is briefly summarized here. For each Zernike mode, the MAL was stepped through a range of coefficient values. At each step, an OCT volume was acquired, and an *en face* image corresponding to the specific depth plane, retinal layer of interest, was extracted. In order to maintain the same retinal layer throughout the optimization process we correct for axial motion during the acquisition by using real time automated retinal tracking software^20^. The image quality metric was calculated based on this *en face* image; for OCT the image intensity is a suitable metric^29^, although other parameters, such as sharpness, could also be used. With the merit function defined as the image intensity, we optimized all the Zernike modes sequentially using a hill climbing coordinate search approach, described in detail in Algorithm 1. For each Zernike mode, we acquired an OCT volume and calculated the merit function value at 11 uniformly sampled coefficient values, and chose the coefficient corresponding to the brightest image to apply to the MAL. The range of coefficient values was determined empirically for each Zernike mode.


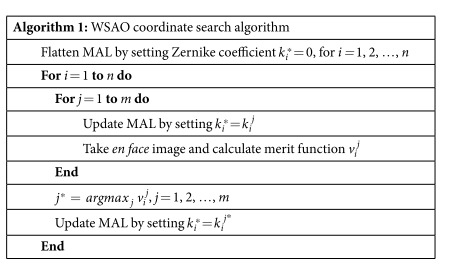


### Real time image acquisition, processing and display

The OCT volume data was acquired, processed and displayed in real time using a custom developed Graphics Processing Unit (GPU) platform that has been previously described^18^,^19^. The GPU processing was integrated with controls for the MAL, VFL, and retinal layer tracking software as well as calculation of the merit function^17^. The user interface was designed to permit dynamic interactive selection of a retinal layer based on the B-scan image, and extract the *en face* view from the OCT volume corresponding to the selected layer. The depth resolved *en face* image was used as the metric for the WSAO aberration correction algorithm. A reduced OCT volume size consisting of 150 × 80 A-scans was acquired at 12.5 frames per second and used for the WSAO optimization process in order to reduce the optimization time.

### Human retinal imaging

The performance of the MAL-WSAO-SS-OCT was demonstrated by imaging the retina of five volunteers free from any remarkable retinal disorder. Human retinal imaging was performed in accordance with the research ethics approved by the Office for Research Ethics (ORE) at Simon Fraser University. Written and informed consent was obtained prior to imaging from all imaging subjects. The probe beam power at the cornea was measured as 700 μW, below the ANSI Maximum Permissible Exposure limits^30^. The probe beam wavelength of 1060 nm with 80 nm FWHM is outside the visible spectrum so that the beam did not cause constriction of the pupil, and subjects with a >5 mm pupil could be readily imaged without mydriasis. In the case of the subjects with a smaller pupil, however, a mydriatic was applied to the subject for inducing dilation of the pupil, which gives the additional benefit of cycloplegia. During retinal imaging, the subject was seated at a table with forehead and chin rest but without a bite bar. The operator aligned the imaging probe beam to the center of the eye pupil under the guidance of a pupil camera that permitted the position of the infrared beam to be observed. The operator adjusted the focus to the retinal layer of interest (e.g. the photoreceptor layer) through interactive control of the VFL with the real time OCT B-scan images used as guidance. Using the retinal tracking software tool, the operator interactively selected the retinal layer at the focus, and activated axial retinal tracking. For the aberration correction of total of 5 Zernike modes, a total optimization time of approximately 3 seconds was required. After finalizing the correction process, the aberration corrected volume data were streamed to hard drive and saved for post processing. Different retina eccentricities were imaged by adjusting the scanning mirror offset and the position of the fixation target.

## Results

Human retinal imaging was performed to demonstrate the functionality and clinical utility of the MAL-WSAO-SS-OCT system. The retinal image was acquired by firstly aligning the fovea approximately at the center of OCT wide-field of view scan covering the transverse area of 1.8 mm (horizontal) × 1.8 mm (vertical) with 400 × 400 A-scans, and then instructing the subject to look on an off-axis fixation target.

**Figure 2:**
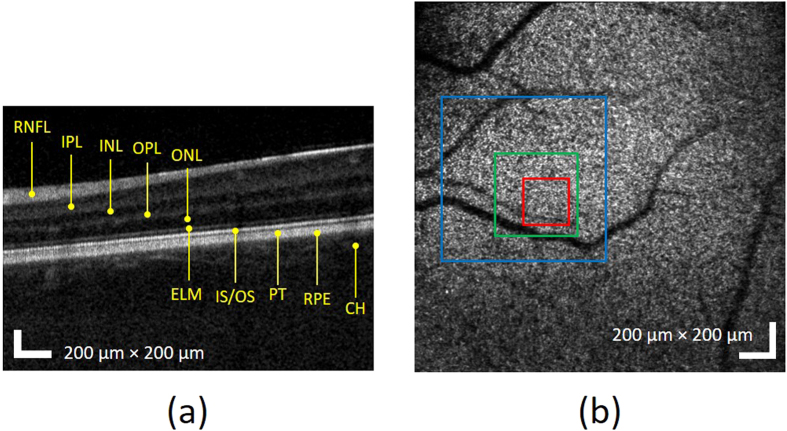
Wide-field of view of OCT (**a**) B-scan and (**b**) en face projection images at IS/OS band before the aberration correction. The red, green, and blue squares represent areas with linear dimensions of 200 μm, 400 μm, and 800 μm, respectively.

Figure 2 shows the wide-field of view OCT B-scan (a) and *en face* projection (b) images without aberration correction. The B-scan image in Fig. 2(a) is presented on a logarithmic intensity scale as is common for commercial (standard resolution) OCT images, enabling visualization of the relevant retinal layers including: the retinal nerve fiber layer (RNFL), internal plexiform layer (IPL), inner nuclear layer (INL), outer plexiform layer (OPL), outer nuclear layer (ONL), external limiting membrane (ELM), junction of the inner and outer segments of the photoreceptor (IS/OS), posterior tip of the outer segment (PT), retinal pigment epithelium (RPE), and choroid (CH). In the OCT *en face* view in Fig. 2(b), morphological characteristics such as shadows casted on photoreceptor layer by retinal vasculature is observed, which is used to guide the imaging location on the retina.

**Figure 3:**
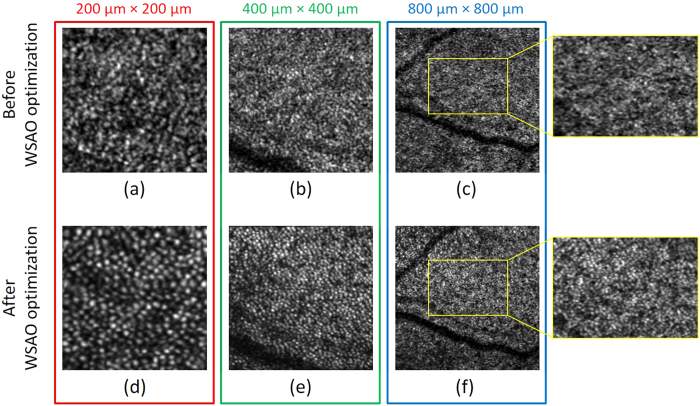
Photoreceptor images before (**a–c**) and after (**d–f**) WSAO aberration correction acquired at three different fields of view: (**a**) and (**d**), 200 μm × 200 μm; (**b**) and (**e**), 400 μm × 400 μm; and (**c**) and (**f**), 800 μm × 800 μm. The data was acquired from the regions marked by the RGB boxes in the wide-field of view en face OCT image in Fig. 2(b).

**Figure 4:**
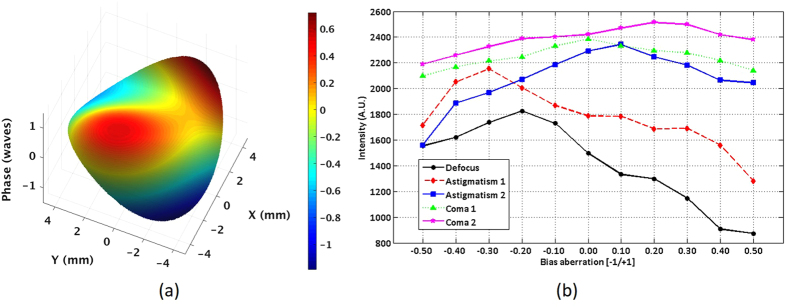
Results of the WSAO optimization process for Fig. 3. (**a**) 3D surface plot of aberrations extracted during the search method and (**b**) the merit function in response to the bias aberrations of the Zernike modes applied to the adaptive lens for wavefront correction. The bias aberration was scaled from [−1.1] based on the maximum amplitude that could be generated by the MAL for each Zernike mode.

To demonstrate the high resolution performance of the system, photoreceptor cone mosaic images were acquired with a sampling density of 200 × 200 A-scans over the transverse areas marked by the RGB windows in the wide-field *en face* OCT image in Fig. 2(b). Figure 3 shows the photoreceptor cone mosaic image results in the three different field of views, in which the top and bottom rows represent the imaging results before and after WSAO aberration correction. The WSAO optimization process was only performed once, followed by the acquisition of the images with varying field of view. Qualitative comparison of the imaging results with and without WSAO aberration correction reveals significant enhancement in the appearance of the cone photoreceptors, identified as resolving each cone with a more circular appearance and higher contrast. Despite the sparse sampling density used for the 800 μm × 800 μm field of view, the cone photoreceptor mosaic could still be resolved at the eccentricity of 7° where the cone size and spacing is much larger than at the fovea. Figure 4 shows the wavefront surface plotted in three dimensions representing aberrations extracted during our search and the merit function recorded at each optimization step. From the merit function plot in Fig. 4(b), the ‘hill climbing’ effect of the WSAO algorithm is clearly observed.

**Figure 5:**
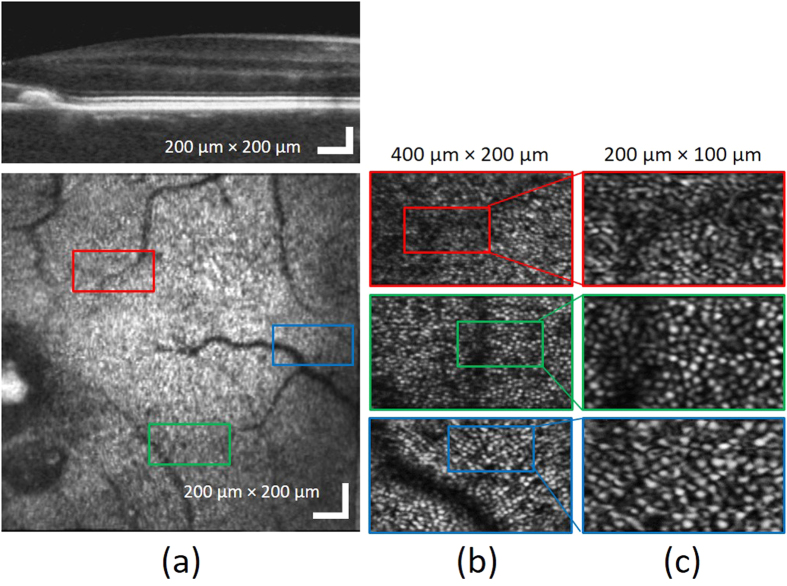
Photoreceptor cone mosaic images acquired at different retinal eccentricities (2.3°, 3.3° and 5.7° ); (**a**) wide-field OCT B-scan and en face images, (**b**) photoreceptor images with 400 μm × 200 μm field of view, (**c**) photoreceptor images with 200 μm × 100 μm field of view.

**Figure 6:**
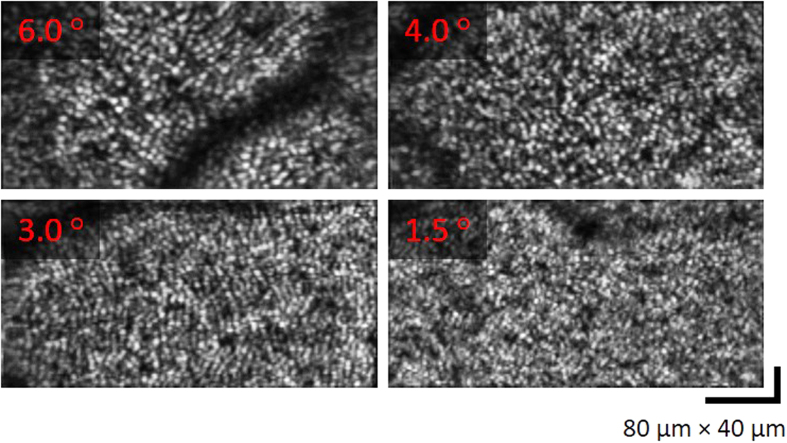
A series of photoreceptor cone mosaic images acquired extracted from OCT Volumes after WSAO correction at different retinal eccentricities. The field of view is 400 μm × 200 μm for all images.

The MAL-WSAO-SS-OCT was used to acquire images of the cone photoreceptor mosaic from a volunteer with a foveal drusen. A wide-field *en face* image of retina is presented in Fig. 5, along with narrow field images revealing the cone mosaic at different retinal eccentricities. In order to reduce motion artifact during imaging, the sampling density was reduced to 200 × 100 A-scans, which increased the volume acquisition rate to ~8 volumes/second acquired, processed, and displayed in real-time. Figure 5(b,c) show the representative photoreceptor imaging results at retinal eccentricities of 2.3° (red), 3.3° (green) and 5.7^o^ (blue). The locations of each image patch in Fig. 5(b) was approximately aligned to the wide-field OCT image by matching the vascular pattern. In a similar way, the appearance of the cone photoreceptor mosaics was used to align the images presented in Fig. 5(c). As expected the cone photoreceptor images acquired at different retinal eccentricities show the variation in cone size and density. In order to identify the limitation of the system resolution, a series of photoreceptor images were acquired on the same subject in Fig. 5 with decreasing eccentricity from the fovea, and the result are presented in Fig. 6. The cone mosaic was resolved at an eccentricity as close as 1.5° where the appearance of the tightly packed cones approaches the calculated system resolution of 2.40 μm based on a numerical aperture of 0.18 in the eye at 1060 nm.

**Figure 7:**
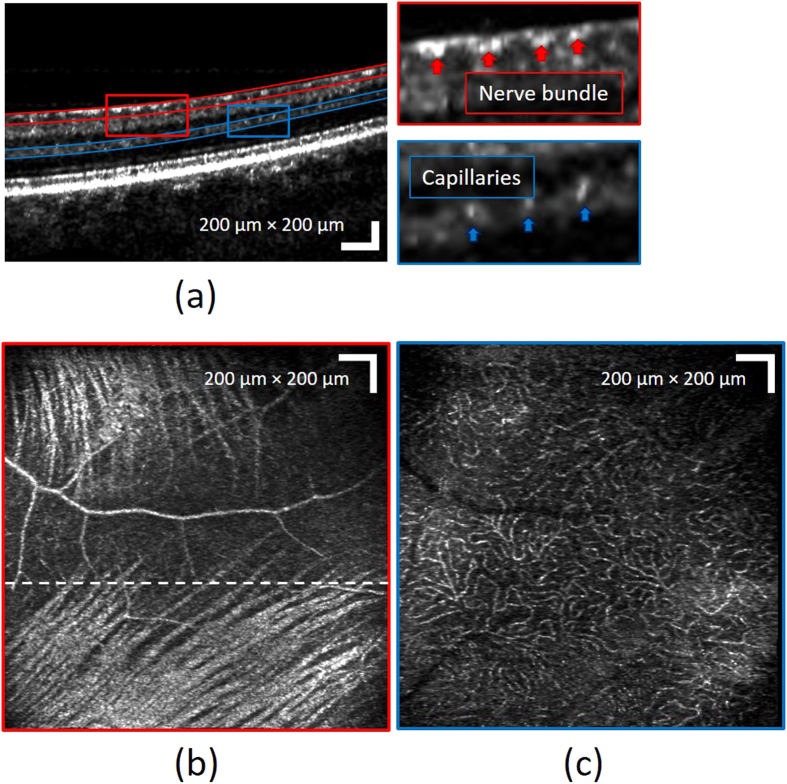
Images of different retinal layers acquired with the MAL-WSAO-SS-OCT. (**a**) The wide-field B-scan image is presented on a logarithmic scale. The red and blue curved lines indicate the depths used for generation of the en face images, (**b**) the retinal nerve fiber layer and (**c**) laminar capillary bed, respectively.

In addition to photoreceptors, different retinal layers could also be visualized using the MAL-WSAO-SS-OCT due to the depth resolved aberration correction capability of the WSAO algorithm. By adjusting the voltage applied to the VFL, the axial position of focal plane of the probe beam could be placed to a specific location in retinal structure, followed by WSAO optimization at that particular depth of focus to enhance resolution of the morphological features. To illustrate the benefit of the depth resolved aberration correction, Fig. 7 shows a result of wide-field of view imaging with the focus set on the RNFL. From the B-scan image in Fig. 7(a), the individual nerve fiber bundles and capillaries are resolved. Figure 7(b,c) are wide-field *en face* images of RNFL and OPL, respectively. In the *en face* images, the nerve fiber bundles in the RNFL and the capillary bed in the OPL are clearly visualized. These retinal imaging results with dynamic depth control demonstrate the versatility of the MAL-WSAO-SS-OCT system in the clinic.

## Discussion

Currently, adaptive optics imaging is not a part of the clinical standard of care for ophthalmologists. In order to achieve a wider adoption of AO in the clinic, ease of operation and robust performance are important for providing meaningful information to the clinicians. In our work, we combined the WSAO algorithm with the 1060 nm SS-OCT engine using two deformable transmissive optical elements. We presented high-resolution photoreceptor mosaic images as well as depth resolved B-scan images with which the clinicians are already familiar. We have also demonstrated the versatility of the system for imaging different retinal layers (e.g. RNFL and OPL). The compact lens-based AO imaging system configuration enabled a relatively simple, small, and robust system that is well suited for clinical imaging.

A characteristic of OCT is that the axial and lateral resolutions are decoupled; the axial resolution is related to the illumination source spectral bandwidth, while the lateral resolution is dependent on NA of imaging optics. Commercially available ophthalmic OCT systems are designed to image the full thickness of the retina, requiring a Rayleigh range on the order of a hundred micrometers and thus a spot size on the order of 20 μm, which is inadequate to resolve the photoreceptors. High resolution OCT combined with conventional AO using a HS-WFS have been reported with similar quality images of the outer retinal layer mosaics as AO-SLO systems^11^,^16^. Importantly, the AO-OCT systems also provide cross-sectional images of the retinal layers that are similar in appearance to the images from the standard resolution systems that are used as a part of current clinical standard. Although the retina is thicker than the Rayleigh range of the AO based systems, the high sensitivity of OCT is adequate to visualize the retinal layers outside the depth of focus, as long as the image depth of the OCT engine is adequate.

We developed our WSAO-OCT imaging system with a commercially available swept source laser operating at 1060 nm wavelength range, unlike the previously reported system based on spectral domain (SD) OCT in the 830 nm wavelength range^28^. The obvious disadvantage of the longer wavelength is that the focused spot size at the retina is ~20% larger for an equivalent imaging NA. However, this is potentially outweighed by the advantages. The longer wavelength is barely visible to the research subject, and is thus less distracting. The longer wavelength has the advantage of being less sensitive to modest scattering in the cornea and lens, which is common in the eyes of patients at ophthalmic clinics. The lower scattering of the 1060 nm light holds promise for improved visibility of features below the retinal pigment epithelium.

The motivations for using the swept source OCT engine with the WSAO are multi-fold. First, high speed wavelength swept lasers are rapidly becoming available, which is important to reduce imaging time and motion artifact. Newly commercially available light sources in the 1060 nm wavelength region operate at depth scan (A-scan) rates up to ~1.5 MHz^31^. As the high speed SS-OCT technology matures, its combination with the lens-based WSAO imaging is complementary. The higher sensitivity of the AO offsets the loss of sensitivity at faster A-scan acquisition rates. Additionally, faster imaging reduces the impact of motion artifact on the optimization algorithm and in the appearance of retinal microstructures such as the photoreceptor mosaic. The wavelength swept lasers have the added benefit that they are less susceptible to so-called phase washout, and potentially permit imaging at sampling rate faster than the A-scan acquisition rate if temporal spectral splitting is applied^32^.

The sample arm delivery optics was configured using lenses (aside from the scanning optics). The lens-based configuration was possible because of the absence of the HS-WFS greatly reduced the system sensitivity to back-reflections from optical elements, additionally OCT rejects any light with optical path length outside of the path lenght of reference arm light. Our lens-based sample arm is simple and small in comparison to mirror based AO setups. The foot-print of the prototype MAL-WSAO-SS-OCT sample arm delivery optics was approximately 18″ × 24″ and was suitable for desk-top operation in a clinical setting. With industrial optical design approaches, the size of the optical system could be readily reduced even further. Because of the limited defocus correction of the MAL used in this report, we incorporated a second element, the VFL. Alternate designs with other deformable elements are also anticipated to function well with WSAO-OCT. Another transmissive adaptive lens that can correct astigmatism has also been described in the literature^33^. The versatility of the WSAO-OCT algorithm could also be used with a reflective deformable element, for example using a large stroke mirror in place of the MAL and VFL; however, we have not yet investigated the performance of this configuration.

Another benefit of the lens-based sample arm configuration is that it is compatible with a wide-field of view in a compact package. The field of view could be further increased through slight changes in configuration from the scanning mirrors to the eye. The field of view that can be imaged with diffraction limited performance is ultimately limited by the isoplanatic patch of the eye, however the capability of imaging relatively large area of the retina (without diffraction limited performance) for navigation on the retina is also important, and it is easily achievable with the lens-based design. A wider field of view on the retina with aberration correction could be obtained by mosaicking multiple acquisitions at different retinal eccentricities. The ability to rapidly run the optimization algorithm is essential for these types of applications.

The results presented in this report demonstrate that the WSAO approach to high resolution retinal imaging is very promising. In addition to the aberration correction while focusing in the outer retina (photoreceptor mosaic), we demonstrated the ability to correct aberrations while focusing on the inner retina directly. Since the image information is used for aberration correction, the anatomical features on which the image-based optimization is performed is always known. This feature also made the WSAO-OCT imaging of the nerve fiber layer straight forward. In contrast, HW-WFS based approaches rely on a backscattered signal from the whole retina, and simply add a defocus term to the aberration correction in order to shift the focal depth axially through the retina. Moreover, the WSAO approach is more favorable for clinically imaging for patients with less ideal optical properties of the eye such as irregular pupil shapes and mild cataracts where getting accurate wavefront aberrations measurement is usually difficult.

The main limitation of the WSAO is the time needed for the algorithm to perform the wavefront correction. With the system presented here, we optimized 5 Zernike modes in ~3 seconds, and patient motion (axial or lateral) during this process would have significant impact on the success of the aberration correction if left uncompensated. It is particularly crucial to correct the axial motion of the subject, as the merit function is extracted from an *en face* projection of a user selected depth range in the OCT B-scan corresponding to a specific retinal layer. The axial tracking algorithm was able to locate the axial position of the retina and maintain the *en face* projection range on the desire retinal layer. Incorporation of lateral tracking would also be very beneficial as demonstrated in recent works^34^,^35^,^36^; in the future, we are planning to add a lateral pupil/retina tracking module to our system. Replacing the light source in this report with a higher repetition rate sweeping laser and the fast-axis galvanometer mounted scanning mirror with a resonant scanner would reduce the convergence time of the WSAO algorithm to less than a second without compromising resolution. A further reduction in correction time could be achieved by acquiring fewer steps per mode and investigating faster converging optimization algorithm^37^,^38^. Another limitation of the WSAO algorithm is that it has very low temporal bandwidth that does not account for accommodation by the intraocular lens. However, cycloplegia (loss of accommodation) is a side effect of mydriasis that is beneficial for the MAL-WSAO-SS-OCT, and dilating drops are safe and commonly used for clinical imaging in patients. In this work, we found that the aberration correction image quality was maintained even after patient blinking, and that tear film dynamics were not a major contributor to image aberration for *in vivo* visualization over the course of seconds.

## Conclusion

The MAL-WSAO-SS-OCT has the potential to help clinicians better understand changes to the retinal microstructure in disease progression preceding irreversible vision loss. The dual adaptive element design, and use of a novel transmissive deformable element contributed to the performance and compact size of the system. The real time display and axial-tracking enabled by the massively parallel processing platform contributed to the clinical readiness of the system. By providing both retinal cross-sectional and *en face* images with cellular resolution, the MAL-WSAO-SS-OCT is well suited to enable a broader investigation of retinal biomarkers of Age-related Macular Degeneration (AMD) such as sub-retinal drusenoid deposits (SDD)^39^. The deep tissue penetration and wide field of view of the MAL-WSAO-SS-OCT also make it a versatile tool for imaging the nerve fiber layer and lamina cribrosa, which are biomarkers in glaucoma. Future application studies with this instrument have the potential to significantly impact the clinical adoption of adaptive optics for high resolution, early stage diagnosis, and monitoring of pathological progression, of diseases causing blindness.

## Additional Information

**How to cite this article**: Jian, Y. *et al*. Lens-based wavefront sensorless adaptive optics swept source OCT. *Sci. Rep.* **6**, 27620; doi: 10.1038/srep27620 (2016).
